# Study on *VEGFA* mRNA delivery via GelMA hydrogel-encapsulated extracellular vesicles for enhanced bone regeneration

**DOI:** 10.1016/j.mtbio.2025.102144

**Published:** 2025-07-28

**Authors:** Shan Li, Yueyang Sheng, Yi You, Ying Wang, Yanzhuo Zhang, Jianfeng Tao, Chengai Wu, Xu Jiang

**Affiliations:** a Department of Molecular Orthopaedics, National Center for Orthopaedics, Beijing Research Institute of Traumatology and Orthopaedics, Beijing Jishuitan Hospital, Capital Medical University, Beijing, China; b Department of Orthopaedics, National Center for Orthopaedics, Beijing Jishuitan Hospital, Capital Medical University, Beijing, China; c Department of Cardiovascular Surgery for the First Affiliated Hospital & Institute for Cardiovascular Science, Suzhou Medical College, Soochow University, Suzhou, China

**Keywords:** Bone defect, *VEGFA* mRNA, Extracellular vesicles, GelMA hydrogel, Bone tissue engineering

## Abstract

Bone regeneration remains a clinical challenge due to the inherent limitations of conventional grafts and synthetic materials. While mRNA-based strategies offer promising therapeutic potential, their clinical application is hindered by systemic delivery barriers and rapid degradation. Here, we present a hybrid extracellular vesicle (EV)-GelMA hydrogel system for the localized delivery of VEGFA mRNA to enhance both angiogenesis and osteogenesis through precise spatiotemporal control. *In vitro,* the VEGFA-EVs-GelMA system significantly enhanced migration and osteogenic differentiation of pre-osteoblastic cells, as evidenced by upregulation of ALP, RUNX2, and OPN expression compared to GelMA controls. In a rat cranial defect model, the VEGFA-EVs-GelMA group showed a marked increase in bone volume/total volume (BV/TV), supported by microcomputed tomography (Micro-CT), at both 4 and 8 weeks post-implantation. Mechanistically, this dual-component platform leverages the biomolecular protection and targeted delivery afforded by EVs, together with the tunable release properties of GelMA hydrogels, to overcome key mRNA delivery barriers. Our results highlight the potential of this system as a clinically translatable strategy for promoting vascularized bone regeneration and provide new insights for the treatment of complex bone defects.

## Graphical abstract

**Figure:**
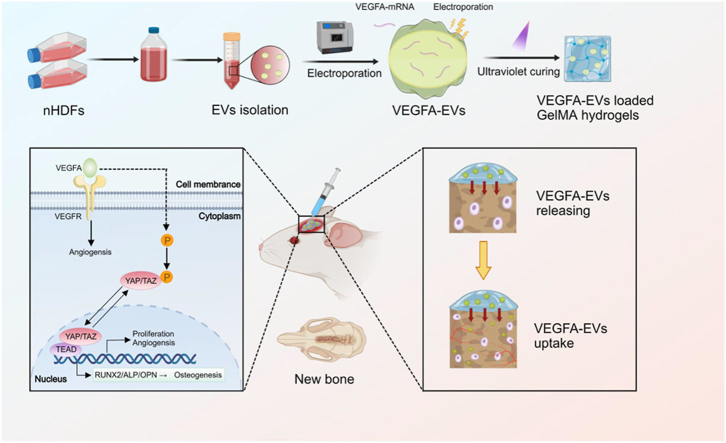

## 1 Introduction

Bone defects caused by trauma, infection, tumor resection, or congenital anomalies, significantly impair skeletal integrity and complicate structural and functional restoration. Such defects not only impose physiological burdens but also profoundly affect patients’ psychological well-being and increase socioeconomic costs for both individuals and healthcare systems [[1], [2], [3], [4]]. Prolonged hospital stays, repeat surgical interventions, and substantial medical expenses further highlight the necessity for innovative therapeutic strategies in bone repair [5,6].

Existing bone reconstruction strategies, including autografts, allografts, metallic implants, and bioceramic scaffolds, exhibit certain clinical efficacies but are often associated with significant shortcomings. Autografts—currently considered the clinical gold standard—demonstrate superior osteoinductivity and osteoconductivity but their utility remains restricted by limited donor availability, donor-site morbidity, and elevated postoperative complications [2,[5], [6], [7], [8]]. Allografts and synthetic materials, while addressing some of these issues, face limitations such as immune rejection, suboptimal biocompatibility, and infection risks, necessitating additional procedures and imposing ongoing economic burdens [[9], [10], [11], [12], [13], [14]]. These limitations underscore the urgent requirement for novel and improved solutions in bone tissue engineering.

Recently, mRNA-based treatment has emerged as a promising strategy in regenerative medicine. In particular, vascular endothelial growth factor A (VEGFA) mRNA stands out for its unique capacity to stimulate angiogenesis, thus significantly enhancing bone regeneration through osteoblast recruitment, differentiation, mineralization and upregulating osteogenic factors such as RUNX2 and COL1A1 [[15], [16], [17], [18], [19], [20], [21]]. Compared to conventional protein-based VEGF delivery, VEGFA mRNA therapy provides transient yet localized expression, reduced immunogenicity, cost-effective biologic production, and controllable and predictable gene translation kinetics, thereby reducing the risks associated with excessive VEGF exposure [17,20]. Nevertheless, clinical translation of VEGFA mRNA remains hindered by its intrinsic instability in physiological environments, inefficient targeted delivery to defect sites, and absence of controlled release methods [20,22].

Extracellular vesicles (EVs), naturally secreted nanoscale entities involved in cell-cell communication by transferring nucleic acids, proteins, and lipids, have become a highly effective class of nano-carriers for therapeutic mRNA delivery [23,24]. EVs provide intrinsic advantages over synthetic nanoparticles, such as superior biocompatibility, stable cargo encapsulation, reduced immunogenicity, and inherent targeting capability, highlighting their clinical potential as VEGFA mRNA delivery vehicles [[10], [11], [12],[25], [26], [27], [28]].

Further complementing EVs-based delivery, gelatin methacryloyl (GelMA) hydrogels provide an optimal biomechanical framework for controlled spatiotemporal release of bioactive compounds. Given their tunable biodegradability, adjustable mechanical properties, and highly porous structures, GelMA hydrogels support cell proliferation, facilitate nutrient diffusion, and enable sustained localized delivery [14,[27], [28], [29], [30], [31]]. Therefore, integrating VEGFA mRNA-loaded EVs within GelMA hydrogels could generate a synergistic delivery platform that harnesses the bioactivity of VEGFA mRNA, EVs-mediated targeted delivery, and GelMA-supported controlled release and structural support.

**Fig. 1:**
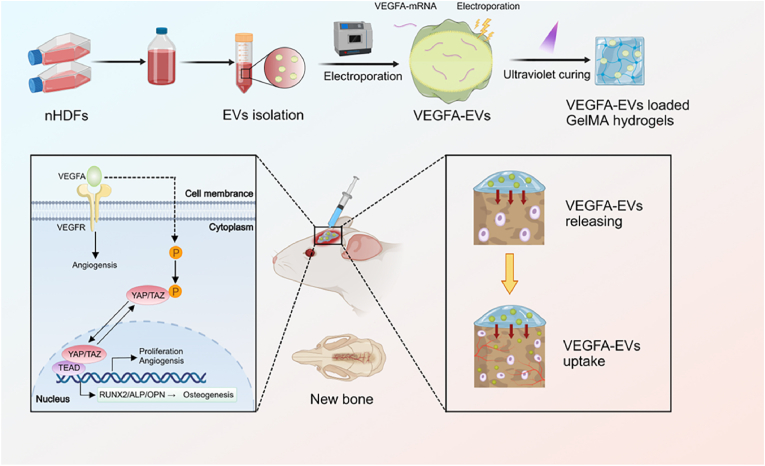
Schematic representation illustrating the delivery of VEGFA mRNA using EVs encapsulated in a biodegradable GelMA hydrogel for in situ bone regeneration.

This study explores the therapeutic efficacy of this integrated VEGFA-EVs-GelMA composite delivery platform. Through a series of in vitro assays, we demonstrate that VEGFA mRNA-loaded EVs significantly enhance osteogenic differentiation and proliferation of MC3T3 pre-osteoblasts. Further, using a rat cranial defect model, we show that VEGFA-EVs-GelMA composites considerably improve bone regeneration and angiogenesis, notably stimulating the formation of H-type vessels and achieving effective integration with surrounding host bone tissues. Collectively, this biomaterial-engineering approach effectively addresses the major barriers to mRNA therapeutic translation and underscores the scalable clinical potential of EV-based mRNA strategies. Ultimately, our findings provide a robust foundation for advancing personalized regenerative therapies tailored specifically for complex bone defect repair (Fig. 1).

## 2 Materials and methods

### 2.1 Reagents and cell culture

Neonatal human dermal fibroblasts (nHDFs, PCS-201-010) were obtained from the American Type Culture Collection (ATCC, Manassas, VA, USA) and cultured in Dulbecco’s Modified Eagle’s Medium (DMEM, Thermo Fisher Scientific, Waltham, MA, USA) supplemented with 10 % heat-inactivated fetal bovine serum (FBS: 10099141C, Thermo Fisher Scientific, Waltham, MA, USA). The cultures were maintained at 37 °C under humidified conditions with 5 % CO_2_.

MC3T3-E1 subclone 14 cells (RRID: CVCL_5437) and specialized Osteogenic Induction Medium (CAS: CM-0378) were procured from (Procell, Wuhan, China). Cells were then grown in complete medium at 37 °C in an incubator with 5 % CO_2_. When they attained 80 %–90 % confluence, they were passaged at a 1:3 ratio. DiI fluorescent dye and *VEGFA* mRNA were procured from Beyotime, China, and APExBIO, Houston, TX, USA, respectively.

### 2.2 Extraction and purification of the EVs

Before isolating EVs, nHDFs were cultured in serum-free Dulbecco’s Modified Eagle’s Medium (DMEM) supplemented with 1 % penicillin-streptomycin for 72 h, as FBS is a known source of exogenous vesicle contamination [23]. The conditioned medium was collected and subjected to initial low-speed centrifugation at 200×*g* for 5 min at 4 °C to remove cells and large debris, followed by medium-speed centrifugation at 2000×*g* for 30 min to eliminate smaller cellular fragments. The supernatant was subsequently filtered through a 0.22 μm filter (Millipore, Bedford, MA, USA) to remove larger particulates. EVs were isolated from the enriched medium using the Total Exosome Isolation Reagent (4478360, Invitrogen, Carlsbad, CA, USA) according to the manufacturer’s protocol. The resulting EV pellet was resuspended in phosphate-buffered saline (PBS, pH 7.4) and stored at −80 °C for later use. To validate the integrity and quality of EVs, multiple characterization assays were performed. Nanoparticle Tracking Analysis (NTA), conducted using the NanoSight NS300 system (Malvern Instruments, Malvern, Worcestershire, UK), confirmed EVs size distribution within the 30–150 nm range and provided quantification of particle concentration. Western blot analysis, probing for EVs-specific protein markers such as CD9, CD63, and TSG101, further verified the purity of isolated EVs. Furthermore, the RNA content and size distribution within the EVs were assessed using the Agilent 2100 Bioanalyzer with the RNA 6000 Pico kit (Agilent Technologies).

### 2.3 Electroporation for mRNA loading

A plasmid containing human *VEGFA* cDNA (NM_000088.3) attached with a green fluorescent protein (GFP) tag (Apexbio) was used for mRNA synthesis. Two billion particles of human dermal fibroblast-derived EVs (nHDF-EVs) were added to Gene Pulser Electroporation Buffer (Bio-Rad) at a 1:9 ratio, which was then added with 10 μg *VEGFA*-GFP mRNA. This preparation was transferred into a 4-mm Gene Pulser/MicroPulser Electroporation Cuvette (Bio-Rad) and subsequently electroporated using a Gene Pulser System (Bio-Rad) under the following conditions: square wave form pulse type (200 V) and 10-ms pulse length at 1-s pulse intervals for 5 pulses. The morphology and characteristic cup-shaped structure of EVs were visualized and confirmed using transmission electron microscopy (TEM) (FEI Talos F200X, Thermo Fisher Scientific, USA). To evaluate the stability of mRNA after loading, a qualitative analysis was performed using reverse transcription-quantitative polymerase chain reaction (qRT-PCR). To confirm the successful delivery of mRNA and its subsequent translation into the target cells, a combination of Western blot analysis and fluorescence microscopy imaging was employed. Specifically, MC3T3 pre-osteoblast cells were incubated under serum-free conditions with VEGFA-mRNA-loaded EVs at a concentration of 10 μg/mL for a duration of 48 h. Following treatment, cellular proteins were extracted, and VEGFA protein expression levels were assessed via Western blot to verify the translation of delivered mRNA. In parallel, fluorescence microscopy was utilized to visualize the GFP-tagged VEGFA protein, and the fluorescence intensity was quantitatively analyzed using ImageJ software to further confirm mRNA delivery efficacy and functional protein production.

### 2.4 Reverse transcription quantitative polymerase chain reaction analysis

The expression of human *VEGFA* mRNA in EVs and osteogenesis-related marker genes involved in this experiment were measured using reverse transcription quantitative polymerase chain reaction (RT-qPCR) following the manufacturer’s recommended protocol. Total RNA was isolated from purified EVs or MC3T3 cells using the MiniBEST Universal RNA Extraction Kit (TaKaRa, China). Subsequently, cDNA synthesis was performed using the Prime Script RT Reagent Kit with gDNA Eraser (Perfect Real Time, TaKaRa, Japan) according to the manufacturer’s instructions. The expression levels of specific target genes were quantified using the SYBR Green I PCR kit (TaKaRa, Japan). Real-time PCR analyses were performed using the Applied Biosystems 7500 Real-Time PCR System (Applied Biosystems, Foster City, CA, USA), with GAPDH as the internal control. The quantification of gene expression was performed using the comparative Ct (2−ΔΔCT) method. The primer sequences used in this study are listed in Table S1.

### 2.5 Characterization of EVs-Loaded GelMA hydrogels

To synthesize GelMA hydrogels (EFL-GM-30, Engineering for Life) loaded with EVs, 1 g GelMA was accurately weighed and dissolved in 10 mL phosphate-buffered saline (PBS) at 60 °C in a water bath while being gently mixed to ensure complete dissolution. After full dissolution, EVs were added to the GelMA solution to achieve a final concentration of 100 μg EV protein/mL GelMA solution, calculated based on the total protein content measured via a bicinchoninic acid (BCA) assay (Thermo Fisher Scientific, Waltham, MA, USA).

Two concentrations of GelMA hydrogels (10 % and 4 % w/v) were prepared in PBS and maintained at 37 °C to ensure complete solubilization prior to further processing. The prepared GelMA solution was subsequently subjected to UV light irradiation to induce cross-linking polymerization and form uniform, transparent EVs-containing hydrogels. A UV cross-linker with a wavelength of 405 nm and an intensity of 20 mW/cm^2^ (model: EFL-1138, Extralink, China) was used for 2 min.

Following cross-linking, pure and EVs-loaded GelMA hydrogels were freeze-dried for 48 h using a laboratory-grade lyophilizer, allowing the samples to reach swelling equilibrium. The freeze-dried hydrogels were subsequently sectioned into uniform slices, coated with a thin layer of gold to enhance conductivity, and examined under a scanning electron microscope (SEM, SU8230, Hitachi, Tokyo, Japan) to characterize the porous microstructure in the cross-sections.

The release kinetics of the EVs from the hydrogels was quantified using a bicinchoninic acid (BCA) assay kit (Thermo Fisher Scientific, Waltham, MA, USA) following the manufacturer’s protocol. The release measurements were conducted in a 24-well plate Transwell system containing 600 μL PBS in the lower chamber and hydrogel samples in the upper chamber. The amount and proportion of EVs released into the PBS over time were quantitatively analyzed.

### 2.6 Rheological testing

The rheological properties of the GelMA and EVs-GelMA hydrogels were evaluated using a rotational rheometer (HAAKE MARS40, Thermo Fisher Scientific, USA) equipped with a parallel-plate configuration. All measurements were conducted at 37 °C to simulate physiological conditions. Shear rate-dependent viscosity was assessed by scanning the shear rate from 1 to 1000 s–1. To investigate the photo-crosslinking behavior, UV irradiation was applied to the samples at t = 30 s, while continuously monitoring the time-dependent changes in storage modulus (G′) and loss modulus (G″). Monitoring G′ and G″ under UV allows for real-time characterization of the sol–gel transition and mechanical stability upon crosslinking. All experiments were performed in triplicate to ensure data reproducibility.

### 2.7 Swelling and degradation testing

Cylindrical hydrogel samples (8 mm in diameter, 6 mm in height) were prepared to evaluate swelling and degradation properties, which are key indicators of hydrogel stability and suitability for tissue engineering. For the swelling assay, the prepared GelMA (or EVs-GelMA) disks were immersed in 2 mL of sterile PBS at 37 °C. At predetermined time points (0, 2, 4, 6, 12, and 24 h), samples were gently blotted with wax paper to remove surface liquid and immediately weighed to determine their swollen weight. The swelling protocol and calculation of the swelling ratio followed the methodology detailed in Ref. [32]. For degradation testing, after equilibration by soaking in PBS at 37 °C for 24 h, the hydrogels were transferred to sterile PBS containing 2 U/mL collagenase type II (Col II) to simulate enzymatic degradation. The samples were collected at 0, 2, 4, 6, and 12 h, blotted, and weighed to evaluate remaining mass. The degradation ratio was determined by comparing the residual weight at each time point to the initial swollen weight, following the procedures in Ref. [32]. These protocols enable quantitative assessment of GelMA hydrogels’ water absorption capacity and enzymatic degradability, which are essential for predicting there in vivo performance as biomaterial scaffolds.

### 2.8 Cell viability assay and live/dead cell staining

Cell viability was assessed using a Cell Counting Kit-8 (CCK-8, Invitrogen, USA). MC3T3 cells were seeded (5000 cells per well) in 96-well plates and incubated for

12 h. Treatments were then applied at 24 and 72 h with 100 μL PBS, 100 μg/mL EVs, 10 % GelMA hydrogel, 100 μg/mL VEGFA-modified EVs (VEGFA-EVs), or 100 μg/mL of combined VEGFA-EVs and GelMA hydrogel (VEGFA–EVs–GelMA). After treatment, the medium was replaced with 10 μL CCK-8 solution per well, followed by incubation for 2 h at 37 °C in the dark. Absorbance at 450 nm was measured using a microplate reader (SpectraMax iD3, Molecular Devices, USA) to determine cell viability, with the control group normalized to 100 %.

For live/dead cell staining, MC3T3 cells were cocultured with treatments in 24-well plates at a cell density of 1 × 10^4^ cells per well for 24 h. Following coculture, 100 μL of staining solution (Abcam: ab65470, USA) was added to each well, and the plates were incubated for 30 min at 37 °C in the dark. Fluorescent images were captured using a fluorescence microscope (Molecular Imaging Devices, Hatwell, GA, USA) and quantitatively analyzed using ImageJ software (National Institutes of Health, Bethesda, MD, USA).

### 2.9 Scratch wound-healing assay

Transfected cells were placed in 6-well culture plates at appropriate densities to ensure confluency. Scratch wounds were carefully created across the diameter of each well using the tips of 200-μL pipettes. Initial photographs of the scratch wounds were taken to establish a baseline. Cell migration was documented immediately at 0 h using a microscope at 10 × magnification. Subsequently, the old medium was removed and replaced with fresh culture medium to support cell recovery and growth. Wound-healing progress was closely monitored by taking photographs at 24 and 72 h after wounding, particularly focusing on the wound line and area of healing. Wound closure was quantified to measure the rate and extent of cell migration and healing. Migration rates were determined using the following formula: Migration Rate = 100 % − (Blank Area Percentage). The initial rates were standardized at 0 % at the 24-h mark.

### 2.10 Alkaline phosphatase and alizarin red staining

MC3T3 cells were seeded in 12-well plates at a density of 2 × 10^4^ cells per well. After 24 h incubation, the initial medium was replaced with osteoinductive medium (Cyagen Biosciences Inc., USA) and replenished every 2–3 days to promote osteogenic differentiation. The rate of osteogenic differentiation was evaluated using the alizarin red staining (ARS; Cyagen Biosciences Inc., Santa Clara, CA, USA) and alkaline phosphatase (ALP) activity assay (Beyotime, Shanghai, China). For the ALP activity assay, MC3T3 cells were treated with [specify treatments, e.g., EVs, VEGFA-EVs, GelMA hydrogels, or control] for 7 days. At the end of the 7-day treatment period, the cells were thoroughly washed four times with PBS and fixed with 4 % paraformaldehyde (PFA) for 15 min. ALP activity was then quantified using the ALP assay kit (Beyotime Biotechnology, Shanghai, China) according to the manufacturer’s instructions.

For the ARS assay, similar initial treatment and washing steps were performed as described for the ALP assay. The cells were fixed with 4 % paraformaldehyde (PFA) for 15 min at room temperature, followed by staining with 2 % alizarin red S (pH 4.1–4.3) for 20 min. Excess stain was gently washed off with PBS to preserve the integrity of the stained mineral deposits. Images of the stained samples were captured using an optical microscope (ImageXpress Micro 4, Molecular Devices, San Jose, CA, USA) to evaluate mineralization, which indicates osteogenic differentiation.

### 2.11 Immunofluorescence staining

MC3T3-E1 cells were subjected to osteogenic induction for 72 h by culturing them in osteogenic differentiation medium consisting of α-minimum essential medium (α-MEM) supplemented with 10 % FBS, 1 % penicillin-streptomycin (P/S; Gibco, Grand Island, NY, USA), 50 μg/mL ascorbic acid, 10 mM β-glycerophosphate, and 100 nM dexamethasone (Sigma-Aldrich, St. Louis, MO, USA). After 72 h of induction, the cells were fixed with 4 % PFA for 15 min at room temperature. Fixed cells were permeabilized with 0.2 % Triton X-100 in PBS for 20 min to facilitate antibody penetration. To reduce nonspecific antibody binding, the cells were blocked with 1 % bovine serum albumin in PBS for 1 h.

After blocking, the cells were incubated with primary antibodies targeting ALP (sc-365765, Santa Cruz Biotechnology), osteopontin (OPN, Proteintech-23418-1-AP), and VEGFA (sc-7269, Santa Cruz Biotechnology) for 2 h at room temperature. After primary antibody incubation, the cells were thoroughly washed with PBS to remove unbound antibodies.

The cells were then incubated with Alexa Fluor 488-conjugated anti-rabbit IgG secondary antibodies (Abcam-ab150077) for 1 h to allow specific detection of the primary antibodies. Subsequently, the cells were stained with 4ʹ,6-diamidino-2-phenylindole (DAPI) to visualize the nuclei.

Images of the final staining results were captured using a high-content screening system (Molecular Imaging Devices, USA), which enabled the detailed visualization and analysis of the protein expression patterns associated with osteogenic differentiation.

### 2.12 Western blotting

Total cellular protein was extracted using radioimmunoprecipitation assay lysis buffer (Keygen Bio Tech, China), according to the manufacturer’s protocol. Protein concentrations were determined using a BCA protein assay kit (Beyotime). Proteins were resolved by sodium dodecyl sulfate–polyacrylamide gel electrophoresis and subsequently transferred to polyvinylidene fluoride membranes. The membranes were blocked using 5 % skim milk for 2 h at room temperature and incubated overnight at 4 °C with primary antibodies specific for ALP (ab307727), RUNX2 (ab76956), OPN (Proteintech-23418-1-AP), Yes-associated protein (YAP; CST-59971), phosphorylated YAP (p-YAP; CST-13008T), transcriptional coactivator with PDZ-binding motif (TAZ; CST-59971), phosphorylated TAZ (p-TAZ; Affinity-AF4316), and β-actin (ab8226). After incubation, the membranes were washed three times with Tris-buffered saline containing 0.1 % Tween® 20 for 6 min each time and subsequently incubated with horseradish peroxidase-conjugated secondary antibodies (mouse IgG- Biorigin-BN20602; rabbit IgG-Biodee-DE0601) at room temperature for 1 h. Protein bands were visualized using an enhanced chemiluminescence detection system (Sage Creation). Quantitative analysis of the protein bands was performed using ImageJ software.

### 2.13 Animal model

Animal experiments were conducted in accordance with ethical standards and were approved by the Animal Care and Use Committee of Beijing Jishuitan Hospital affiliated with the Capital Medical University. A total of sixty (n = 60) six-week-old male Sprague–Dawley (SD) rats were procured from Charles River (Beijing, China) to ensure adequate sample size and facilitate robust statistical analyses. To evaluate the effectiveness of GelMA hydrogels loaded with VEGFA-EVs in promoting bone regeneration, a critical-sized cranial defect (8 mm in diameter) was surgically created on the parietal bone of each animal under sterile conditions. Animals were anesthetized using isoflurane inhalation (2 % in oxygen), and standardized cranial defects were carefully drilled using a trephine bur, avoiding injury to the dura mater. Hemostasis was achieved using sterile gauze. The study period was divided in 4- and 8-week treatment cycles. Following surgery, in each treatment cycle, the animals were randomly allocated into five experimental groups (n = 6 per group) using a random number table method to minimize selection bias and ensure balanced group distribution. The groups and their treatments were as follows: (1) Control: Received 100 μL sterile PBS, (2) EVs: treated with 100 μL EV, 1000 μg/mL, (3) GelMA hydrogel: treated with 100 μL of 10 % GelMA hydrogel, (4) EVs-GelMA: treated with 100 μL of 10 % GelMA hydrogel containing EVs (1000 μg/mL), and (5) VEGFA–EVs–GelMA: treated with 100 μL of 10 % GelMA hydrogel containing VEGFA-EVs (1000 μg/mL). The respective treatment solutions were pre-gelled and administered via syringe into the cranial defect site, ensuring uniform filling of the entire defect area and close contact with adjacent bone tissue. The surgical site was then carefully sutured to prevent infection and ensure proper recovery. Rats were housed individually under controlled environmental conditions (22 ± 2 °C, 55 % ± 10 % humidity, 12-h light/dark cycle) and provided ad libitum access to food and water. Postsurgical analgesia was provided with meloxicam (2 mg/kg, subcutaneous injection) every 24 h for 3 days.

At four or eight weeks postoperatively, the animals were humanely euthanized via carbon dioxide inhalation. The cranial bone specimens were carefully harvested and fixed in 4 % paraformaldehyde (PFA) at 4 °C for subsequent analysis. Bone regeneration outcomes were systematically assessed using dual-energy X-ray absorptiometry (DEXA) to evaluate bone mineral density, micro-computed tomography (micro-CT; SkyScan 1176, SkyScan, Aartselaar, Belgium) for three-dimensional structural reconstruction, and immunohistochemistry for the identification of osteogenic and angiogenic markers.

### 2.14 DEXA evaluation of specimens

Body composition analysis was conducted on all rats using iNSiGHT VET DEXA (OsteoSys, Korea). Each rat was weighed before scanning. The rats were positioned in a prone position on the scanner bed, with the limbs and tail fully extended. The machine was operated between 60 and 80 kV with a tube current of 0.8 mA. The total scanning time was 25 s, including an X-ray exposure time of 10 s. The scanner generated data on the bone mineral density (g/cm^2^) based on the attenuation of energy levels. The region of interest (ROI) was manually selected, and automated calculations were performed within the iNSiGHT software to produce volumetric bone mineral density values.

### 2.15 Micro-CT analysis

A cylindrical ROI with an 8-mm radius was delineated to calculate the new bone volume. Three-dimensional reconstruction was subsequently performed using the system-specific software. Bone volume fraction (BV/TV), open porosity, trabecular number (Tb. N), trabecular thickness (Tb. Th), trabecular separation (Tb. Sp), and total bone volume (BV) were analyzed.

### 2.16 Histological analysis of osteogenic and angiogenic markers

Specimens were decalcified in 10 % EDTA for 3 weeks, embedded in paraffin, and sliced into 5-μm sections. The sections were stained with hematoxylin and eosin (HE) and Masson’s trichrome to evaluate bone and collagen regeneration. Immunohistochemistry was performed using osteogenic markers such as RUNX2 (ab76956, Abcam), OCN (23418-1-AP, Proteintech), ALP (ab307727, Abcam), and COL1a (GB11022, Servicebio). Immunofluorescence was performed with angiogenic markers OPN (ab63856, Abcam), OCN (23418-1-AP, Proteintech), ALP (sc-365765, Santa Cruz), and CD31 (3528S, CST). DAPI staining (C1002, Beyotime) was performed to label nuclei. Images were captured with an inverted fluorescence microscope (Pannoramic 250FLASH). Fluorescent signals from CD31, ALP, OPN, and OCN were quantified over three ROIs for each 20x image covering both the bone defect and newly regenerated bone areas. Measurements were performed three times and averaged using ImageJ software. DAPI co-staining was performed to normalize cell counts in each ROI, enabling the calculation of the percentage of positively stained areas. Four weeks post-implantation, heart, liver, spleen, lung, and kidney samples were collected for toxicity assessment via HE staining.

### 2.17 Statistical analysis

Data are expressed as mean ± standard deviation (SD). Statistical analyses were performed using GraphPad Prism 9.0 software (GraphPad Software, San Diego, CA, USA). One-way analysis of variance (ANOVA) was performed to compare differences across groups. A p-value ≤0.05 was considered statistically significant. Statistical significance in tables and figures is denoted as follows: "NS" indicates not significant (p > 0.05); "∗" indicates p ≤ 0.05; "∗∗" indicates p ≤ 0.01; and "∗∗∗" indicates p ≤ 0.001. These notations are used in the captions and/or legends of the respective tables and figures to indicate the level of statistical significance between specific experimental groups.

## 3 Results

### 3.1 Preparation and delivery of VEGFA mRNA within EVs

**Fig. 2:**
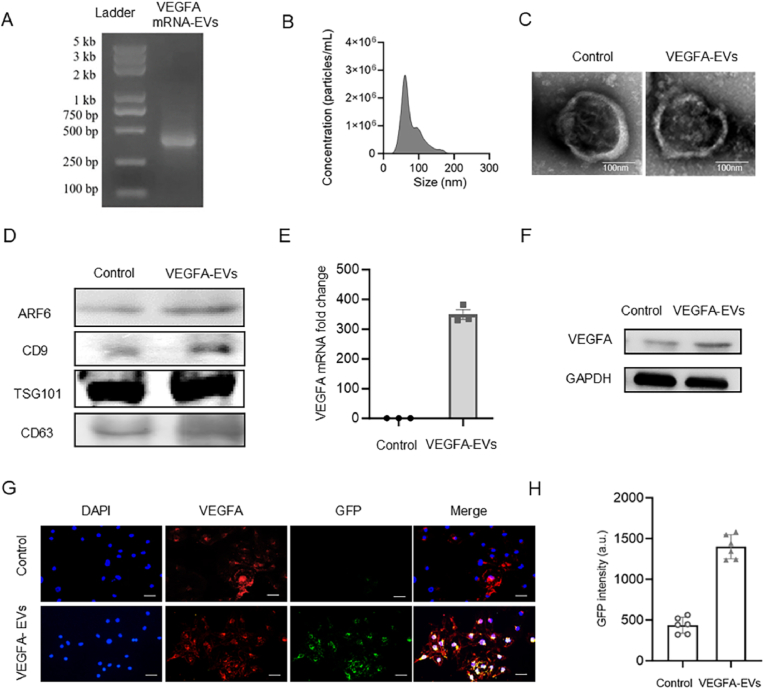
Construction and characterization of EV-mediated VEGFA mRNA delivery. A. Gel electrophoresis analysis of total RNA extracted from 3.78x10^8^ EVs generated via electroporation with the VGEFA-GFP plasmid. B. Nanoparticle tracking analysis (NTA) for particle size distribution and concentration of EVs. C. Transmission electron microscopy (TEM) images of EVs and VEGFA-EVs to examine their ultrastructural microscopy. D. Western blot analysis of EV-specific markers (ARF6, CD9, TSG101, CD63) in isolated EVs and VEGFA mRNA-EVs. E. RT-qPCR analysis demonstrating significantly enhanced mRNAs transcription in VEGFA mRNA-loaded EVs compared to control EVs over a 24 h period. F. Western blot analysis showing increased VEGFA protein levels in MC3T3 cells treated with VEGFA-EVs compared to controls. G. Fluorescence microscopy images of serum-starved MC3T3 cells treated with VEGFA-GFP EVs, confirming the protein translation of delivered VEGFA-GFP mRNA after 48 h. Nuclei were stained with DAPI; and untreated EVs served as the negative control. Scale bar, 100 μm. H. Quantitative fluorescence intensity of MC3T3 cells treated with VEGFA-EVs after 48 h, with results presented as mean ± SD (n = 3; ∗∗∗P < 0.001, Control vs VEGFA-EVs).

EVs were successfully isolated from the culture supernatants of nHDFs. Assessment of agarose gels using the Bioanalyzer (Agilent 2100, Agilent Technologies, Santa Clara, CA, USA) confirmed that the full length of the transcribed *VEGFA* mRNA was approximately 420 base pairs long (Fig. 2A). VEGFA mRNA was efficiently encapsulated within the EVs using a standardized bulk electroporation (BEP) technique, as previously documented [33]. The characterization of EVs was performed using nanoparticle tracking analysis to determine their size distribution and transmission electron microscopy (TEM) to examine their morphology. TEM analysis demonstrated that both native and *VEGFA* mRNA-loaded EVs exhibited a round, cup-shaped morphology, while nanoparticle tracking analysis confirmed a consistent size distribution (30–150 nm) in both groups (Fig. 2B and C). These findings indicate that the encapsulation process did not alter the structural integrity or morphology of the EVs.

Further molecular characterization using western blotting revealed the expression of conventional microvesicle markers, including CD9, CD63, and TSG101, in both the control and *VEGFA* mRNA-loaded EV groups (Fig. 2D). Notably, ARF6, a key molecule involved in EVs biogenesis, was also detected in both groups. These findings indicate that the loading of *VEGFA* mRNA did not alter the intrinsic biological properties of the EVs (Fig. 2D). To assess the efficacy of *VEGFA* mRNA loading, RT-qPCR analysis revealed that the *VEGFA* mRNA in loaded EVs was significantly elevated compared to the control EVs (approximately 350-fold increase) (p < 0.001, Fig. 2E). Delivery efficacy was evaluated in vitro by treating MC3T3 cells with VEGFA-GFP EVs or free VEGFA-GFP for 48 h under serum-starvation conditions. Western blotting results revealed a significant increase in VEGFA protein levels in MC3T3 cells treated with VEGFA-GFP EVs (Fig. 2F). Additionally, fluorescence microscopy analysis demonstrated markedly elevated fluorescence intensity in the VEGFA-GFP EVs group compared to cells treated with free VEGFA-GFP. Specifically, VEGFA mRNA-loaded EVs exhibited an approximately 35-fold increase in VEGFA protein levels compared to the control EVs (Fig. 2G and H).

### 3.2 Characterization of GelMA hydrogels loaded with EVs

**Fig. 3:**
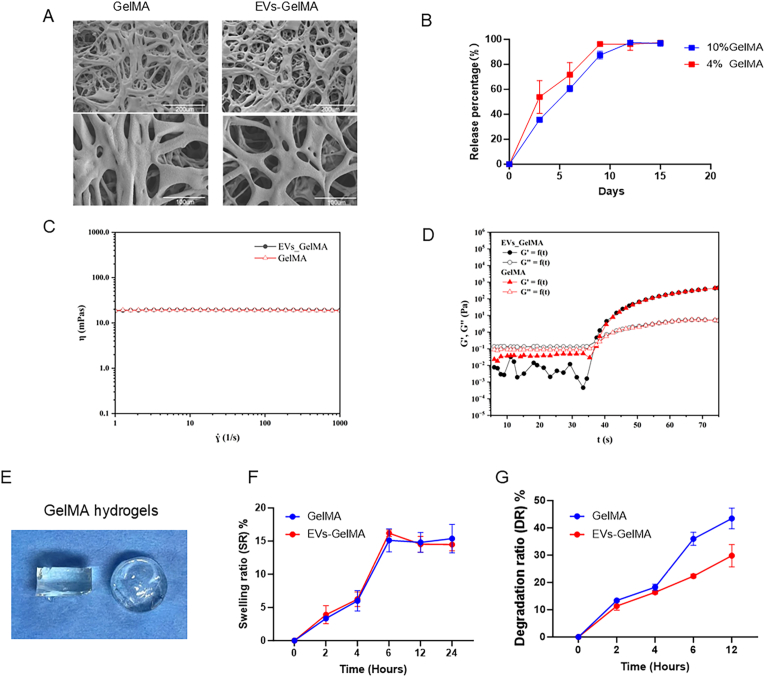
Characterization and biocompatibility evaluation of EV-loaded GelMA hydrogels. A. Scanning electron microscopy (SEM) images highlighting the microstructures of pure GelMA hydrogels and EV-loaded GelMA hydrogels. Scale bar, 200 (up) and 100 μm (down). (n = 3; GelMA vs EVs-loaded GelMA hydrogels). B. In vitro release kinetics of EVs from composite GelMA hydrogels at different concentrations. (n = 3 for all groups). C. Shear thinning behavior of GelMA and EVs-GelMA at 37 °C temperature. D. The photosensitivity of GelMA and EVs-GelMA. E. Representative photographs of the hydrogel morphologies are provided as insets. F. Swelling ratio (SR) performance of GelMA and EVs-GelMA. G. In vitro degradation ratio (DR) of GelMA and EVs-GelMA.

SEM revealed that the GelMA hydrogels exhibited a well-defined porous architecture, facilitating improved intercellular communication and efficient nutrient diffusion (Fig. 3A). Notably, after encapsulating EVs, SEM images showed that the hydrogel’s intrinsic pore structure was preserved, indicating that the EV-loading process did not alter the fundamental microstructure. This interconnected porosity is critical for enabling sustained release of EVs and preserving the bioactivity of encapsulated therapeutic cargo. To evaluate the release dynamics, the cumulative release profiles of EVs from GelMA hydrogels were monitored for both 4 % and 10 % concentrations (Fig. 3B). Release profiles showed a near-linear increase over the initial 9 days, followed by a plateau phase, indicative of a sustained and controlled release. Statistical analysis revealed no significant differences in cumulative release percentages between the 4 % and 10 % GelMA hydrogels (p > 0.05), suggesting that the matrix concentration does not markedly influence the release kinetics within this tested range. Rheological properties were assessed by measuring viscosity as a function of shear rate (Fig. 3C). Both GelMA and EVs-GelMA hydrogels displayed nearly Newtonian behavior, with viscosity remaining stable and largely independent of shear rate. Importantly, the incorporation of EVs had minimal effect on the overall viscosity, indicating that EV encapsulation does not impair the hydrogel’s injectability or processability. Further, dynamic oscillatory rheology investigations assessed the storage modulus (G′) and loss modulus (G″) before and after EV loading (Fig. 3D). Both G′ and G″ values exhibited only minor changes upon EV encapsulation, confirming that the mechanical integrity and elasticity of the hydrogels are maintained. UV-triggered gelation was characterized by a rapid increase in both moduli, and the presence of EVs did not affect the sol–gel transition response to ultraviolet exposure. Swelling and degradation characteristics were evaluated to further elucidate the hydrogel’s physical properties. Representative photographs of the hydrogel morphologies are provided as insets (Fig. 3E). As shown in Fig. 3F, both GelMA and EVs-GelMA hydrogels achieved swelling equilibrium within 6–8 h, with no significant difference observed between the two groups. Degradation assays (Fig. 3G) revealed that while both hydrogels progressively lost mass over time, the incorporation of EVs modestly slowed the initial degradation rate during the first 12 h, possibly due to stabilizing interactions between EVs and the hydrogel matrix. Collectively, these results confirm that GelMA hydrogels provide a robust and stable matrix for EV encapsulation, offering favorable microstructural, mechanical, and degradation profiles well-suited for biomedical applications. Importantly, the encapsulation of EVs does not compromise the hydrogel’s structural integrity, while enabling efficient and sustained EV release—highlighting the promise of this platform for the controlled delivery of bioactive vesicles in regenerative medicine and tissue engineering contexts.

### 3.3 Effect of GelMA hydrogels loaded with VEGFA-EVs on cell migration

**Fig. 4:**
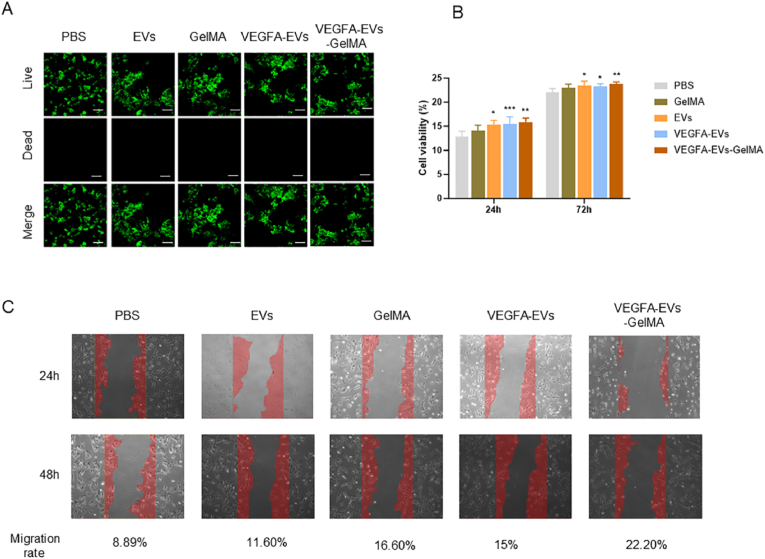
Effects of VEGFA-EV-incorporated GelMA hydrogels on cellular behavior. A. Live/Dead staining of cells after 24 h of co-culture in a 24-well plate, followed by a 72 h treatment with 100 μL of different agents. Live cells fluoresced green, while dead cells fluoresced red. Fluorescent images were captured using a fluorescence microscope. Scale bar, 100 = μm (n = 3 for all groups). B. Cytotoxicity analysis using the CCK-8 assay at 24 and 72 h of co-culture with different treatment agents. Results are expressed as mean ± SD, with statistical significance determined via one-way ANOVA and two-tailed Student’s t-test (∗P < 0.05, ∗∗P < 0.01 and ∗∗∗P < 0.001 compared with PBS group). C. Representative images from a scratch assay at 24 and 48 h with quantitative analysis of cell migration. Migration rate was calculated using the formula: Migration Rate = 100 % - Blank Area Percentage, normalized to 0 % at the initial time point.

To assess the effects of VEGFA-EVs-loaded GelMA hydrogels on cellular behavior, MC3T3 cells were treated with five experimental groups: PBS (control), EVs, 10 % GelMA, VEGFA-EVs, and VEGFA–EVs–10 % GelMA. Live/dead staining revealed that most cells remained viable (green fluorescence), indicating minimal cytotoxicity from either the EVs or GelMA hydrogels (Fig. 4A). The cytotoxicity of the treatments was evaluated using CCK-8 assays at 24 and 72 h of coculture. No significant toxic effects on MC3T3 cells were observed for any of the treatment groups, as evidenced by the absence of a reduction in cell viability compared to the PBS control group (Fig. 4B). Importantly, the evaluation showed that treatments with EVs, VEGFA-EVs, and VEGFA–EVs–GelMA significantly enhanced cell proliferation at both time points, with VEGFA–EVs–GelMA exhibiting the most pronounced effect. At 72 h, the VEGFA–EVs–GelMA group demonstrated a significantly higher proliferation rate compared to the PBS control group (p < 0.01) and showed a more significant difference compared to the EVs and VEGFA-EVs groups. This significant promotion in cell proliferation supports the hypothesis that VEGFA-loaded EVs embedded in GelMA hydrogels provide a synergistic effect, substantiating their superior capacity to enhance osteoblast activity.

The scratch assay results demonstrated significant enhancement in the migration of MC3T3 cells toward the wound area in the EVs, GelMA, VEGFA-EVs, and VEGFA–EVs–GelMA treatment groups. The migration rates increased by 11.6 %, 16.6 %, 15 %, and 22.2 %, respectively, at 48 h. Notably, the VEGFA–EVs–GelMA treatment group demonstrated the most enhanced migration capacity (Fig. 4C).

### 3.4 Enhanced osteogenic differentiation through *VEGFA* mRNA delivery via 10 % GelMA hydrogel-embedded EVs in vitro

**Fig. 5:**
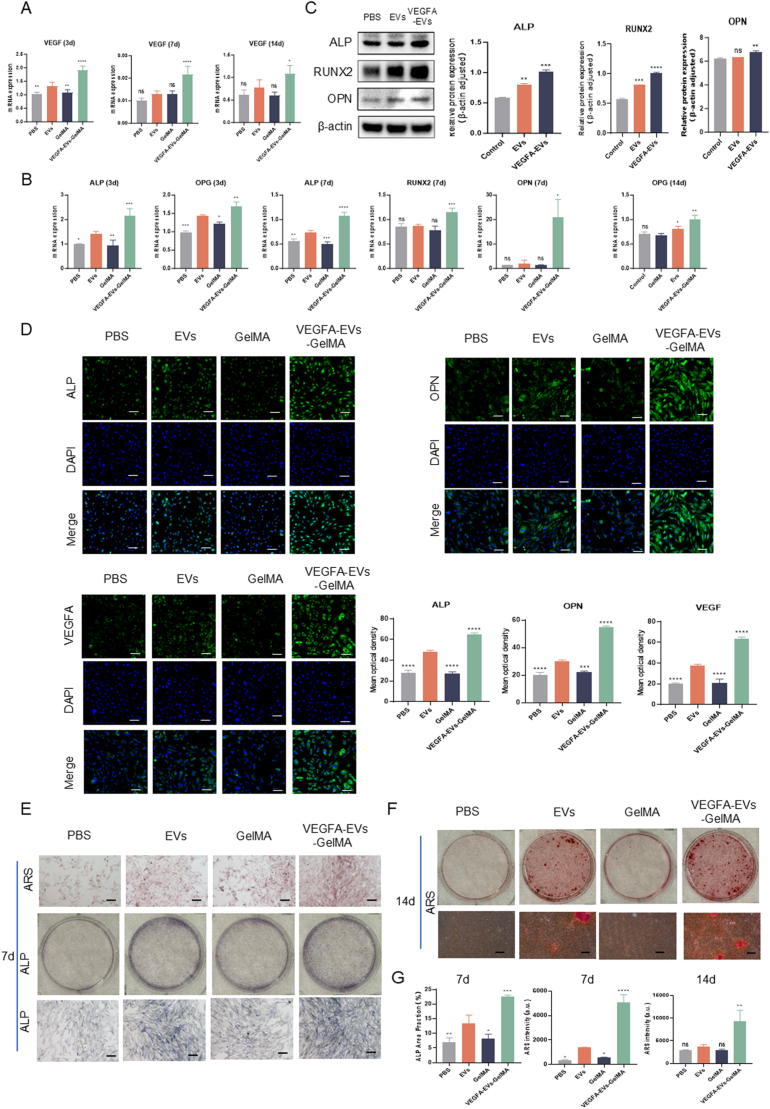
Regulation of osteogenic differentiation in MC3T3 cells by VEGFA-EVs. A. Real-time PCR quantification of VGEFA expression in the co-culture system at 3, 7, and 14 days. B. Real-time PCR of osteogenesis-related genes expression (ALP, OPG, RUNX2, and OPN) in MC3T3 cells over the same time points. C. Protein expression analysis of osteogenic markers (ALP, RUNX2, and OPN) using Western blot, with β-actin as the loading control. Quantitative analyses of ALP, RUNX2, and OPN protein levels are also shown. D. Immunofluorescence staining and corresponding quantitative analysis of osteogenic differentiation markers (OPN and ALP) and VEGFA at three days. Scale bar = 100 μm. E-F. Visualization of alkaline phosphatase (ALP) activity and mineralizing matrix formation by alizarin red staining after 7 and 14 days of co-culture. (E: Scale bar = 200 μm, F: Scale bar = 500 μm) G. Quantitative analysis of ARS staining and ALP staining in included. All data are presented as mean ± SD (n ≥ 3; ∗P < 0.05, ∗∗P < 0.01 and ∗∗∗P < 0.001 compared with PBS control group).

We then evaluated and confirmed in vitro the effects of *VEGFA* mRNA delivery via EVs embedded in hydrogels on the induction of osteogenic differentiation. For this assessment, four experimental groups were established, all cocultured with MC3T3 cells: PBS (control), EVs, GelMA, and VEGFA–EVs–GelMA. RT-qPCR results revealed that VEGFA expression levels in the VEGFA–EVs–GelMA treatment group were significantly elevated compared with those in the other groups after 3, 7, and 14 days of coculture (Fig. 5A). Relative mRNA expression levels of key osteogenic markers ALP, OPG, Runx2, and OPN assessed using RT-qPCR on days 3, 7, and 14, were significantly elevated in the VEGFA–EVs–GelMA treatment group compared with those in the other groups (Fig. 5B). Furthermore, western blotting results revealed increased expression levels of ALP, Runx2, and OPN in the VEGFA–EVs–GelMA hydrogel group compared with those in the other groups (Fig. 5C). Consistent with these findings, immunofluorescence staining revealed significantly higher fluorescence signals for ALP, OPN, and VEGF in the VEGFA–EVs–GelMA hydrogel group, further validating the promotion of osteogenesis by this treatment (Fig. 5D).

On day 7 of coculture, ARS staining revealed a significantly larger calcification area in the VEGFA–EVs–GelMA group compared with that seen in the control group, indicating enhanced mineral deposition (Fig. 5E). ALP staining results confirmed the presence of osteoblasts in all groups, with a notable increase in osteogenic activity in the VEGFA–EVs–GelMA group (Fig. 5E). On day 14 of coculture, MC3T3 cells treated with VEGFA–EVs–GelMA exhibited a marked increase in mineralized matrix formation compared with that in the other treatment groups (Fig. 5F). The quantification of ARS staining and ALP staining on day 7 and 14 displayed in Fig. 5G.

### 3.5 Activation of YAP/TAZ signaling by VEGFA-enriched EVs in MC3T3 cells

**Fig. 6:**
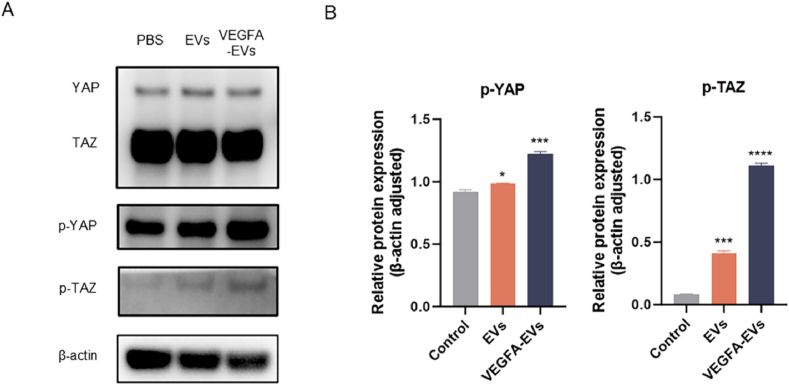
Regulation of YAP/TAZ signaling by VEGFA-EVs in MC3T3 cells. A. Western blotting for YAP, TAZ, phosphorylated YAP (p-YAP), and phosphorylated TAZ (p-TAZ), with β-actin as a loading control. B. Quantitative analysis of p-YAP and p-TAZ levels. Results are expressed as mean ± SD, with statistical significance determined by one-way ANOVA and two-tailed Student’s t-test (∗P < 0.05, ∗∗P < 0.01 and ∗∗∗P < 0.001 compared to the PBS control group).

We subsequently investigated the underlying molecular mechanisms of the osteoinductive properties of the engineered EVs. Previous studies have found that VEGFA promotes bone formation and angiogenesis through the phosphorylation-mediated activation of the YAP/TAZ signaling pathways [15]. Western blot analysis revealed a significant increase in YAP/TAZ phosphorylation in both the EVs and VEGFA-EVs groups compared with the PBS control group. Notably, the VEGFA-EVs group exhibited a markedly greater osteoinductive effect than the EVs-only group (Fig. 6A and B), demonstrating the critical role of YAP/TAZ activation in osteogenesis and angiogenesis. This differential activation indicates the key molecular pathways by which VEGFA-enriched EVs may enhance osteogenic and angiogenic responses, providing insights into the mechanism of action of advanced skeletal tissue engineering.

### 3.6 VEGFA-EVs-loaded GelMA hydrogels enhanced the retention of VEGFA-EVs in vivo

**Fig. 7:**
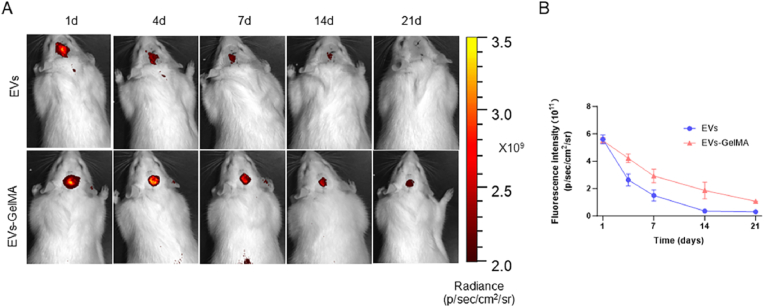
VEGFA-EVs-Loading into GelMA Hydrogels Enhances VEGFA-EVs Retention *In Vivo*. A. Fluorescence imaging demonstration sustained release of Dil-labeled EVs from GelMA Hydrogels in a rat cranial defect model. Representative in vivo imaging system (IVIS) images illustrate EV distribution over 21 days in rats injected with either free Dil-labeled EVs or GelMA-EVs formulations. B. Quantitative fluorescence intensity of retained EVs over time, presented as mean ± SD (n = 3).

We investigated the ability of GelMA hydrogels for sustained release of VEGFA-EVs in the subdural space of rat skulls. In vivo imaging revealed that EVs remained consistently present within the GelMA hydrogel for up to 3 weeks without a significant reduction in signal intensity. In contrast, the PBS group exhibited a rapid decrease in the EVs signal by day 14. These findings demonstrate that the GelMA hydrogels significantly retained VEGFA-EVs, indicating their potential for prolonged therapeutic effects (Fig. 7A and B).

### 3.7 In vivo bone regeneration using VEGFA-loaded EVs in GelMA hydrogels

**Fig. 8:**
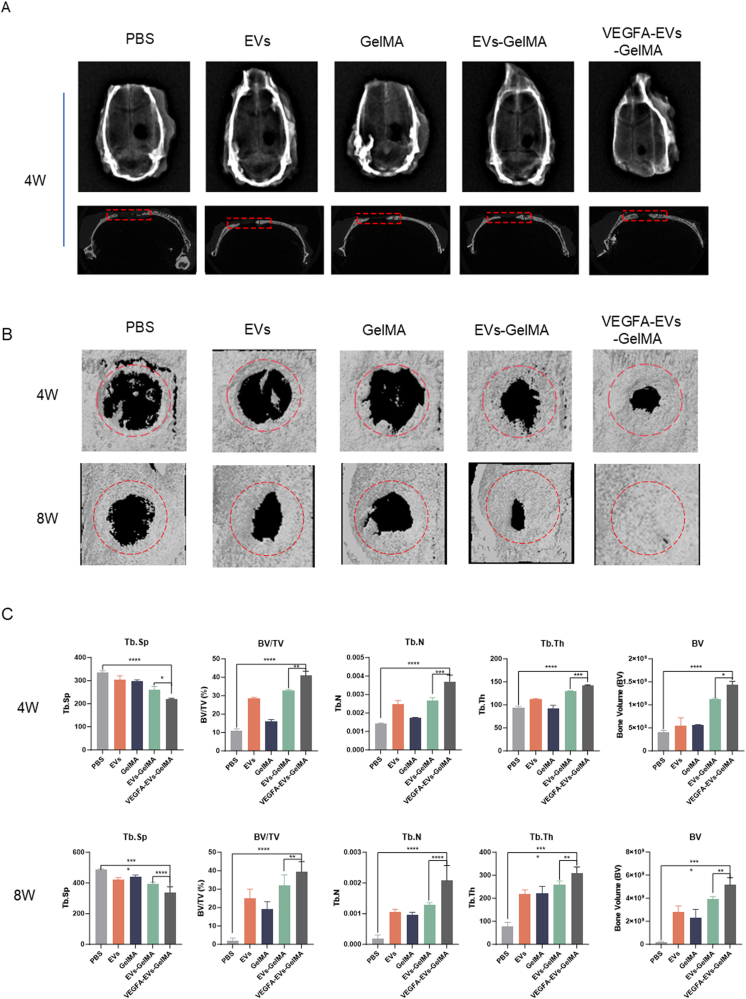
In vivo evaluation of bone regeneration capability of VEGFA–EVs–loaded GelMA hydrogel. A. X-ray imaging of cranial defects sites treated with various formulations. B. Three-dimensional reconstructions of regenerated bone in treated groups using micro-CT imaging at 4 and 8 weeks. C. Micro-CT quantitative analysis of bone volume and density at 4 and 8 weeks, expressed as mean ± SD (n = 5; ∗p < 0.05; ∗∗p < 0.01; ∗∗∗p < 0.001).

The regenerative efficacy of VEGFA-loaded EVs embedded within GelMA hydrogels was evaluated for in situ bone healing using DEXA, micro-CT, immunohistochemistry, and immunofluorescence. A rat cranial defect model was established, and various treatments were administered. Then, the hydrogels were crosslinked in situ using UV light. At 4 and 8 weeks post-treatment, rat cranium samples were collected for assessment. X-ray imaging and micro-CT analysis revealed substantial bone regeneration in rats treated with VEGFA-EVs-GelMA hydrogels, as demonstrated by a marked reduction in the defect area compared with the control group at both time points (Fig. 8A and B). Notably, the degree of bone regeneration exhibited a time-dependent increase, with more pronounced bone formation and remodeling observed at 8 weeks. Furthermore, quantitative analysis showed that BV/TV, Tb. N, Tb. Th, and BV were significantly higher, while Tb. Sp was significantly lower, in the VEGFA-EVs-GelMA hydrogel treatment group compared to the control group. Moreover, these parameters showed further improvement at 8 weeks compared to 4 weeks (Fig. 8C).

**Fig. 9:**
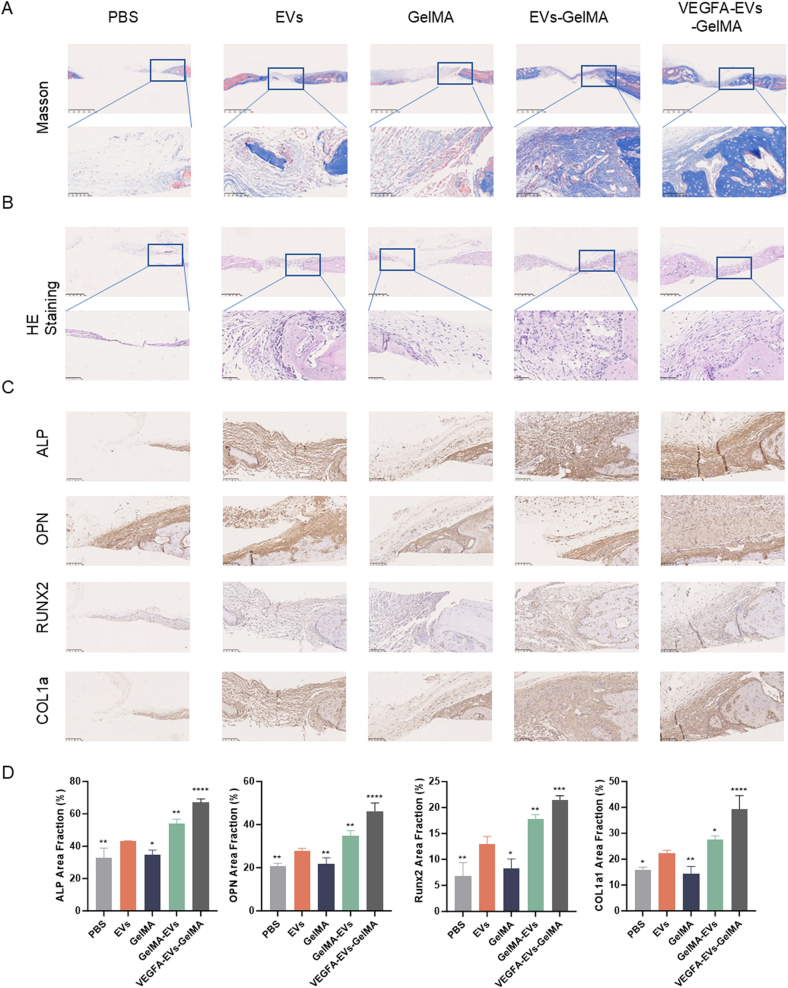
Histological assessment of bone regeneration within GelMA hydrogel-treated defects. A. Hematoxylin and eosin (HE) staining of rat cranial defect sites at four weeks post-treatment. Scale bar = 500 μm; magnified image, scale bar = 100 μm. B. Masson’s trichrome staining at four weeks post-surgery visualizing collagen deposition and tissue organization. Scale bar = 500 μm; magnified image, scale bar = 100 μm. C. Immunohistochemical analysis of osteogenesis-related proteins (COL1A1, ALP, OPN, and RUNX2) in bone tissue at four weeks post-surgery. Scale bar = 100 μm. D. Quantitative analysis of positive-stained areas for each protein marker. Significance is indicated as ∗p < 0.05; ∗∗p < 0.01; ∗∗∗p < 0.001.

Histological analyses using HE and Masson’s trichrome staining revealed a substantial proliferation of new bone tissue, as characterized by distinct pink and blue staining primarily observed in the VEGFA-EVs-GelMA-treated group compared with that seen in the control group (Fig. 9A). Moreover, HE staining revealed a significant increase in bone tissue formation 4 weeks post-treatment in the VEGFA-EVs-GelMA group (Fig. 9B). Immunofluorescence assays revealed elevated levels of the osteogenic proteins ALP, OCN, RUNX2, and COL1a in the VEGFA-EVs-GelMA hydrogel group relative to the other groups (Fig. 9C and D).

**Fig. 10:**
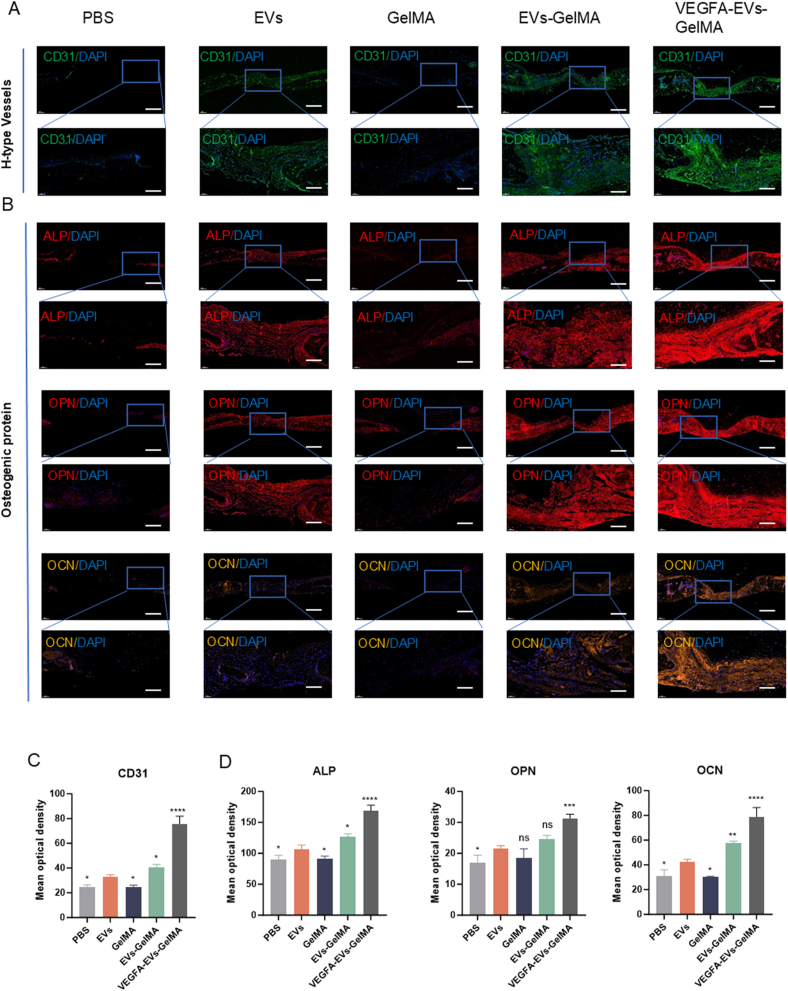
Immunofluorescence analysis of osteogenic and vascular differentiation markers in bone tissue at four weeks. A. Immunofluorescence staining for proteins associated with H-type vessels. Scale bar = 500 μm; magnified image, scale bar = 100 μm. B. Immunofluorescence staining of osteogenic markers (ALP, OPN, and OCN) in regenerated bone. Scale bar = 500 μm; magnified image, scale bar = 100 μm. **C**. Semiquantitative analysis of H-type vessel related proteins. Scale bar = 100 μm. D. Semiquantitative analysis of osteogenic differentiation markers (ALP, OPN, and OCN). Data are presented as mean ± SD (n = 5; ∗p < 0.05; ∗∗p < 0.01; ∗∗∗p < 0.001).

CD31 immunohistochemical staining, described in detail in the methods section, revealed a significant presence of vascular endothelial cells in the VEGFA–EVs–GelMA hydrogel group. This finding indicates the formation of osteogenic–angiogenic H-type vessels, which play a crucial role in coupling angiogenesis and osteogenesis (Fig. 10A and C). Corresponding quantitative analysis demonstrated significantly increased vascularization in the VEGFA–EVs–GelMA group compared with the other groups (Fig. 10B and D). These results suggest that the VEGFA–EVs–GelMA hydrogel creates an enhanced regenerative environment by promoting both angiogenesis and osteogenesis. The presence of these H-type vessels highlights the dual role of the hydrogel in stimulating bone formation and vascular integration, critical for tissue regeneration.

To assess the biosafety of these engineered EVs in potential clinical applications, toxicity evaluations of VEGFA-loaded GelMA hydrogels were conducted across major rat organs. These results revealed no significant morphological changes in the heart, liver, spleen, lungs, and kidneys compared with those in the control group (Fig. S1), confirming the non-toxicity of the VEGFA-loaded GelMA hydrogel. These findings reveal the potential of the sustained delivery of osteo-functionalized EVs within a biodegradable hydrogel platform as a promising cell-free therapeutic approach for in situ bone regeneration.

## 4 Discussion

Personalized bone repair methods are a critical area of focus in the development of bone tissue engineering techniques. The advent of precision medicine indicates the significant potential of personalized therapies that address individual patient requirements, laying a reliable foundation for the development of efficient and customized treatment plans [34,35].

In this study, we demonstrated that the EVs-GelMA composite system provides a highly effective platform for the targeted delivery of VEGFA mRNA, leveraging dual protection and release mechanisms to ensure sustained bioactivity. Specifically, EVs function as endogenous carriers [[10], [11], [12],^31^,^36^], encapsulating VEGFA mRNA and thereby shielding it from nuclease-mediated degradation in extracellular environments. Transmission electron microscopy (TEM) and nanoparticle tracking analysis (NTA) confirmed the structural integrity and bioactivity of VEGFA mRNA-loaded EVs in vitro. This encapsulation strategy markedly increased mRNA stability and bioavailability relative to free mRNA, as evidenced by a 350-fold enrichment of VEGFA mRNA within EVs compared to control groups (Fig. 2E), underscoring the efficiency of EV-mediated delivery.

The inclusion of GelMA hydrogels afforded additional spatiotemporal regulation, with the material’s high porosity and tunable biomechanical properties forming a favorable milieu for EV immobilization [6,[27], [28], [29]]. This configuration enabled gradual, controlled release of encapsulated VEGFA mRNA over 15 days (Fig. 3B), with equilibrium between hydrogel stability and timely release evidenced by in vitro assays. Importantly, encapsulation of EVs did not significantly affect the hydrogel’s rheological or degradation properties**,** confirming the suitability of the system for sustained delivery applications (Fig. 3C–G).

Critically, the dual-stage release strategy—comprising initial EVs encapsulation of mRNA followed by GelMA-mediated spatiotemporal control—was validated by the sustained, stepwise liberation of VEGFA mRNA over 15 days (Fig. 3B and F). This coordinated release directly translated to improved local bioavailability and therapeutic efficacy, supporting an extended window for osteogenesis and angiogenesis.

*In vivo*, the VEGFA-EVs-GelMA composite demonstrated robust bone regeneration and vascularization following cranial defect implantation in rats. At both 4 and 8 weeks, micro-CT and histological analyses revealed significant increases in BV/TV, Tb. N, Tb. Th, and BV, with concomitant reductions in Tb. Sp, indicative of enhanced tissue maturation (Fig. 8, Fig. 9). These data underscore the composite’s capacity to support both early and prolonged bone regeneration—crucial for functional repair.

Mechanistically, the therapeutic benefits of the system are mediated by the activation of the Hippo signaling pathway, particularly through VEGFA mRNA-induced phosphorylation of YAP and TAZ. Quantitative protein analyses demonstrated significantly elevated levels of p-YAP and p-TAZ in MC3T3 cells treated with VEGFA-EVs (Fig. 6A and B). As YAP/TAZ signaling orchestrates endothelial behavior and osteogenic transcriptional regulation (e.g., via RUNX2), these findings reinforce the integral role of this molecular axis in linking angiogenesis and osteogenesis [[37], [38], [39]]. Upregulation of osteogenic markers (RUNX2, ALP, OPN, COL1A1) was further validated in vitro and in vivo (Fig. 5, Fig. 9), providing direct evidence for molecular coupling in bone regeneration.

Compared with conventional methods, this study introduces several innovations. Unlike approaches utilizing direct VEGF protein delivery or gene therapy, VEGFA mRNA offers transient, controllable expression with lower immunogenicity risks. Likewise, GelMA hydrogels outperform traditional scaffolds—such as polylactic acid or bioceramics—in mechanical tunability, biocompatibility, and microenvironmental mimicry [[27], [28], [29], [30],^40^]. The described composite thus addresses multiple tissue engineering challenges, including precision targeting, prolonged release, and optimized signaling crosstalk.

The selection of neonatal human dermal fibroblasts (nHDFs)-derived EVs is also noteworthy, as these vesicles confer superior biocompatibility and immunotolerance [25], reflected in the absence of adverse immune responses in implanted animal models. Compared to mesenchymal stem cells (MSCs)-derived EVs, which may prompt variable immunogenicity, nHDF-EVs offer greater consistency and safety. Furthermore, the electroporation loading strategy adopted here far surpassed passive loading approaches in both encapsulation efficiency and preservation of EV integrity, as demonstrated by fluorescence imaging and functional readouts (Fig. 2F–H).

Despite these advances, challenges remain. Further long-term in vivo studies are required to assess the mechanical robustness and clinical reliability of the regenerated tissue beyond 8 weeks. Standardization in EVs production, purification, and loading is necessary to address variability and facilitate translational scalability. Additionally, continued development of GelMA derivatives with enhanced strength could further enable applications in load-bearing bone defects.

In conclusion, the interaction of VEGFA mRNA, EVs, and GelMA hydrogels results in a sophisticated, synergistic platform for orchestrated angiogenesis and osteogenesis, via precisely controlled mRNA delivery and downstream molecular activation. The sustained functional outcomes observed at 8 weeks reinforce this system’s potential for progressive, long-term bone repair. Collectively, our findings extend current understanding of gene delivery architecture and provide a blueprint for future translation to precision bone repair therapies.

## 5 Conclusion

This study demonstrated that VEGFA–EVs–GelMA hydrogels exhibit dual regenerative functionality by concurrently promoting osteogenesis and angiogenesis—notably through H-type vessels formation and upregulation of osteogenic markers. This next-generation composite system enhanced cell viability, proliferation, lineage-specific differentiation, and vascular integration, underlining its promise as a versatile biomaterial for bone tissue engineering. Moving forward, attention to manufacturing scalability, mechanical optimization, and deeper mechanistic elucidation will be crucial for clinical translation.

## CRediT authorship contribution statement

**Shan Li:** Writing – original draft, Project administration, Methodology, Investigation, Data curation, Conceptualization. **Yueyang Sheng:** Writing – original draft, Validation, Methodology, Data curation. **Yi You:** Writing – original draft, Methodology, Conceptualization. **Ying Wang:** Investigation. **Yanzhuo Zhang:** Methodology. **Jianfeng Tao:** Methodology. **Chengai Wu:** Writing – review & editing, Project administration, Investigation, Funding acquisition, Conceptualization. **Xu Jiang:** Writing – review & editing, Project administration, Funding acquisition, Conceptualization.

## Ethics approval statement

The animal studies were meticulously supervised and received approval from the Animal Care and Use Committee of Beijing Jishuitan Hospital affiliated with Capital Medical University.

## Funding statement

This research was supported by the 10.13039/501100004826Beijing Natural Science Foundation (Grant No.7244286), the 10.13039/501100005088Beijing Municipal Health Commission (BJRITO-RDP), and the Beijing Natural Science Foundation-Haidian Original Innovation Joint Fund (Grant No. L222089).

## Declaration of competing interest

We declare that we have no financial and personal relationships with other people or organizations that can inappropriately influence our work, there is no professional or other personal interest of any nature or kind in any product, service and/or company that could be construed as influencing the position presented in, or the review of, the manuscript entitled.

## Data Availability

Data will be made available on request.

## Appendix A Supplementary data

Multimedia component 1 Multimedia component 1

Fig. S1. Hematoxylin and eosin staining of major organs. Comprehensive histological examination of the heart, liver, spleen, lung and kidneys across different experimental groups. Scale bar = 100 μm.Multimedia component 2
