# Supplementary material for: Study on VEGFA mRNA delivery via GelMA hydrogel-encapsulated extracellular vesicles for enhanced bone regeneration
Source: Mater Today Bio. 2025 Jul 28;34:102144. doi: 10.1016/j.mtbio.2025.102144 (PMC12337882; doi:10.1016/j.mtbio.2025.102144)
Supplement: Multimedia component 1 [file mmc1.docx]

Table S1. Sequences of primers used in qRT-PCR.

| Genes |  | Sequences (5'---3') |  |
| --- | --- | --- | --- |
| VEGF  RUNX2  OCN  OPN  BSP  OSX  OPG  ALP  GAPDH | Forward  Reverse  Forward  Reverse  Forward  Reverse  Forward  Reverse  Forward  Reverse  Forward  Reverse  Forward  Reverse  Forward  Reverse  Forward  Reverse | GTGAGGTGTGTATAGATGTGGGG  ACGTCTTGCTGAGGTAACCTG  AGAGTCAGATTACAGATCCCAGG  TGGCTCTTCTTACTGAGAGAGG  ATGGCACCACCGTTTAGGG  CTGGACTTTATTTTGGAGCAGC GACAGTCGAATCCATAGCAGC  AGAGACGACCGCATAAGGAAT  GCTTTAATGAGAAGTGTGCTTGG  TCAGGGTCCTTCGATCTTCAG  GGAAAGGAGGCACAAAGAAGC  CCCCTTAGGCACTAGGAGC  TTCAACGAAACGCCCATCAAT  AGTTCCGAAGTGTTCACCCTG  CAGCGGGTAGGAAGCAGTTTC  CCCTGCACCTCATCCCTGA  AATGGATTTGGACGCATTGGT  TTTGCACTGGTACGTGTTGAT | |
