# Supplementary figures and images for: Study on VEGFA mRNA delivery via GelMA hydrogel-encapsulated extracellular vesicles for enhanced bone regeneration
Source: Mater Today Bio. 2025 Jul 28;34:102144. doi: 10.1016/j.mtbio.2025.102144 (PMC12337882; doi:10.1016/j.mtbio.2025.102144)

**
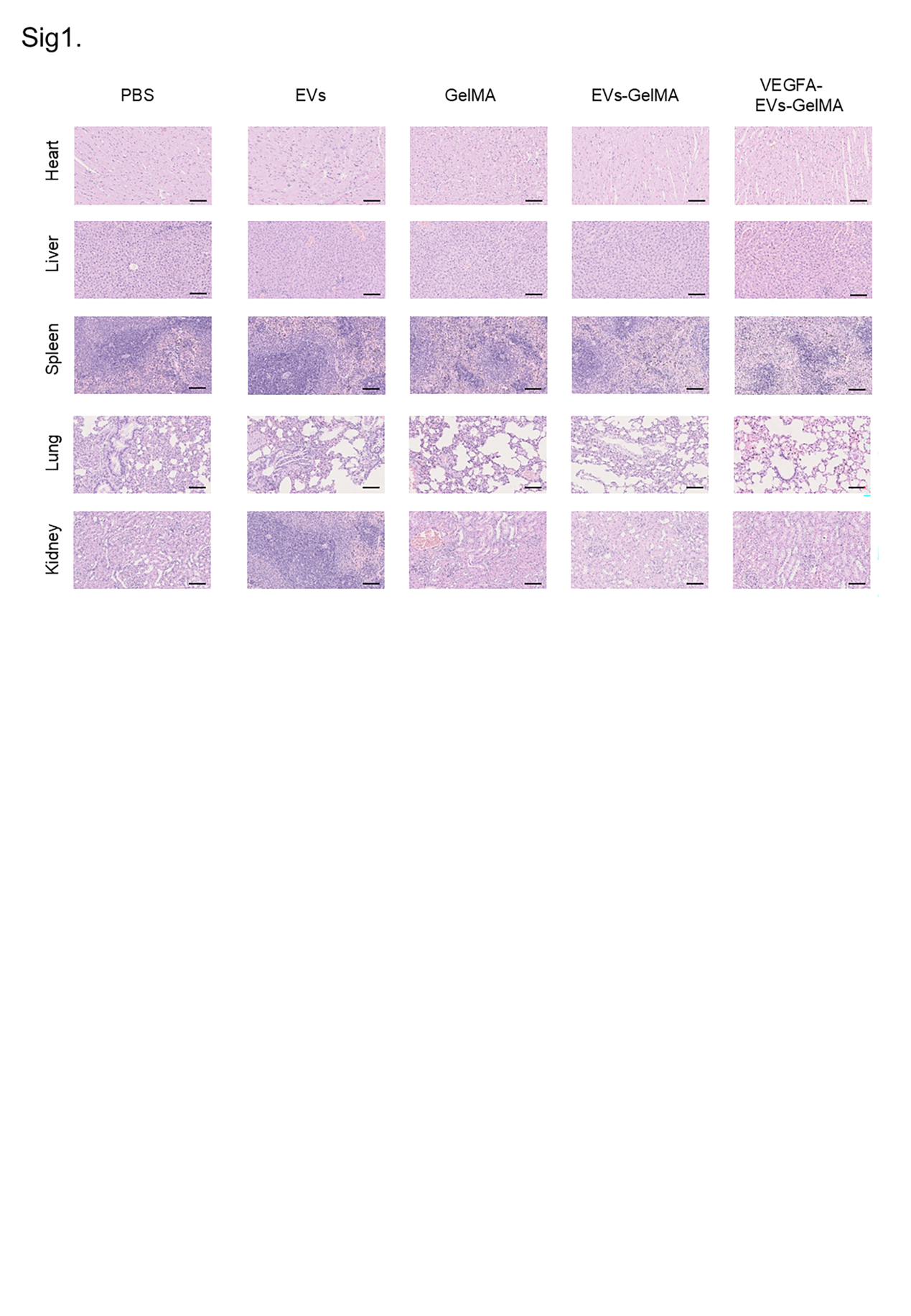
**

Supplement: Supplementary file 2 — Fig. S1. Hematoxylin and eosin staining of major organs. Comprehensive histological examination of the heart, liver, spleen, lung and kidneys across different experimental groups. Scale bar = 100 μm.Multimedia component 2 [file mmc2.docx]
